# Suppression of β-catenin/TCF transcriptional activity and colon tumor cell growth by dual inhibition of PDE5 and 10

**DOI:** 10.18632/oncotarget.4741

**Published:** 2015-07-25

**Authors:** Nan Li, Xi Chen, Bing Zhu, Verónica Ramírez-Alcántara, Joshua C. Canzoneri, Kevin Lee, Sara Sigler, Bernard Gary, Yonghe Li, Wei Zhang, Mary P. Moyer, E. Alan Salter, Andrzej Wierzbicki, Adam B. Keeton, Gary A. Piazza

**Affiliations:** ^1^ Department of Biochemistry and Molecular Genetics, The University of Alabama at Birmingham, Birmingham, Alabama, USA; ^2^ Drug Discovery Research Center, Mitchell Cancer Institute, University of South Alabama, Mobile, Alabama, USA; ^3^ Drug Discovery Division, Southern Research, Birmingham, Alabama, USA; ^4^ INCELL Corporation LLC, San Antonio, Texas, USA; ^5^ Department of Chemistry, University of South Alabama, Mobile, Alabama, USA

**Keywords:** colorectal cancer, sulindac, PDE5, PDE10, β-catenin

## Abstract

Previous studies suggest the anti-inflammatory drug, sulindac inhibits tumorigenesis by a COX independent mechanism involving cGMP PDE inhibition. Here we report that the cGMP PDE isozymes, PDE5 and 10, are elevated in colon tumor cells compared with normal colonocytes, and that inhibitors and siRNAs can selectively suppress colon tumor cell growth. Combined treatment with inhibitors or dual knockdown suppresses tumor cell growth to a greater extent than inhibition from either isozyme alone. A novel sulindac derivative, ADT-094 was designed to lack COX-1/-2 inhibitory activity but have improved potency to inhibit PDE5 and 10. ADT-094 displayed >500 fold higher potency to inhibit colon tumor cell growth compared with sulindac by activating cGMP/PKG signaling to suppress proliferation and induce apoptosis. Combined inhibition of PDE5 and 10 by treatment with ADT-094, PDE isozyme-selective inhibitors, or by siRNA knockdown also suppresses β-catenin, TCF transcriptional activity, and the levels of downstream targets, cyclin D1 and survivin. These results suggest that dual inhibition of PDE5 and 10 represents novel strategy for developing potent and selective anticancer drugs.

## INTRODUCTION

Colorectal cancer (CRC) is the 3rd most common malignant disease in the Western world and is a major public health problem that accounts for up to 12% of all newly diagnosed cancers in the United States [1]. The incidence and mortality rate of CRC continues to decline because of endoscopic screening that allows for the early detection and removal of precancerous adenomas. However, approximately 40–50% of CRC patients who undergo surgery, chemotherapy or radiation ultimately relapse and die from metastatic disease [2]. As such, there is an unmet medical need for safe and effective chemopreventive drugs, especially for individuals at high risk of developing CRC who present with precancerous lesions or have been diagnosed with malignant disease and treated, but remain at risk for disease recurrence.

Epidemiological studies have reported that the long-term use of non-steroidal anti-inflammatory drugs (NSAIDs) can significantly reduce the incidence and risk of death from colorectal cancer by as much as 50–60% [3]. Certain prescription-strength NSAIDs such as sulindac have also been reported to cause regression of precancerous adenomas in individuals with familial adenomatous polyposis (FAP) [4]. These observations are consistent with studies in rodent models reporting that NSAIDs strongly inhibit intestinal tumorigenesis induced by chemical carcinogens or by mutations in the *APC* gene [5, 6]. However, the risk of gastrointestinal, renal, and cardiovascular toxicity associated with COX-1 or COX-2 inhibition and suppression of physiological prostaglandins limits the long-term use of NSAIDs for chemoprevention [7].

While the pharmacological basis for the antineoplastic activity of NSAIDs is commonly attributed to COX-2 inhibition, many investigators have concluded that other mechanisms account for their tumor growth inhibitory activity, mostly because higher concentrations are generally required to inhibit tumor cell growth compared with concentrations required to inhibit COX-2 [8, 9]. As evidence for a COX-independent mechanism, the non-COX inhibitory sulfone metabolite of sulindac was reported to inhibit the growth of various tumor cell lines *in vitro* and suppress tumorigenesis in multiple animal models [10]. The mechanism by which sulindac sulfone inhibits tumor cell growth may involve cyclic guanosine monophosphate phosphodiesterase (cGMP PDE) inhibition based on its ability to inhibit certain cGMP PDE isozymes at concentrations that suppress tumor cell growth and ability of certain cGMP PDE inhibitors to also suppress tumor cell growth by a similar mechanism involving the suppression of β-catenin signaling [11, 12]. More recently, the COX inhibitory sulfide metabolite of sulindac (SS) and other NSAIDs, including the COX-2 selective inhibitor, celecoxib, have also been reported to inhibit cGMP PDE activity at concentrations that inhibit tumor cell growth [13, 14].

Cyclic nucleotide PDEs are a superfamily of related phosphohydrolases that selectively catalyze the hydrolysis of the 3′ cyclic phosphate bonds in adenosine and/or guanosine 3′, 5′ cyclic monophosphate (cAMP and/or cGMP). Up to 11 PDE isozyme families comprising at least 21 different isoforms have thus far been identified that display different substrate specificity, biochemical regulatory properties, pharmacological sensitivity, as well as tissue distribution patterns [15]. PDE1, 2, 3, 10 and 11 are dual substrate-degrading isozymes, while PDE5, 6, 9 are selective for cGMP, and PDE4, 7 and 8 are cAMP selective. PDE functions in the cell to terminate cyclic nucleotide signaling, whereby inhibition blocks degradation, resulting in the elevation of intracellular cyclic nucleotide levels to amplify the duration and/or magnitude of the signal to activate various downstream mediators, such as cyclic nucleotide-dependent protein kinases, PKA and PKG [16].

The cGMP-specific PDE5 appears to be an important target of sulindac that is overexpressed in colon, breast, and lung tumors [13, 14, 17–19]. However, the involvement of additional cGMP degrading isozymes could not be ruled out, given the non-selective cGMP PDE inhibitory activity of sulindac and the modest tumor cell growth inhibitory activity of PDE5 specific inhibitors, such as sildenafil [13, 14, 19, 20]. We recently reported that PDE10 is overexpressed in colon tumors cells and essential for their growth [21]. Similar to PDE5, inhibition of PDE10 can selectively inhibit colon tumor cell growth by activating the cGMP/PKG pathway to suppress β-catenin-dependent TCF transcriptional activity. Here we show that: 1) PDE5 and 10 are elevated in colon tumor cells compared with normal colonocytes, 2) inhibitors or siRNA knockdown of PDE5 and 10 can selectively inhibit colon tumor cell growth, and 3) dual inhibition is more effective than inhibiting either isozyme alone. We also characterize a novel, non-COX inhibitory sulindac derivative, referred to as ADT-094 that potently and selectivity inhibits colon tumor cell growth by inhibiting PDE5 and 10 and activating cGMP/PKG signaling to suppress β-catenin/TCF-transcriptional activity, resulting in cell cycle arrest and apoptosis induction.

## RESULTS

### PDE5 and 10 inhibition suppresses colon tumor cell growth

**Figure 1:**
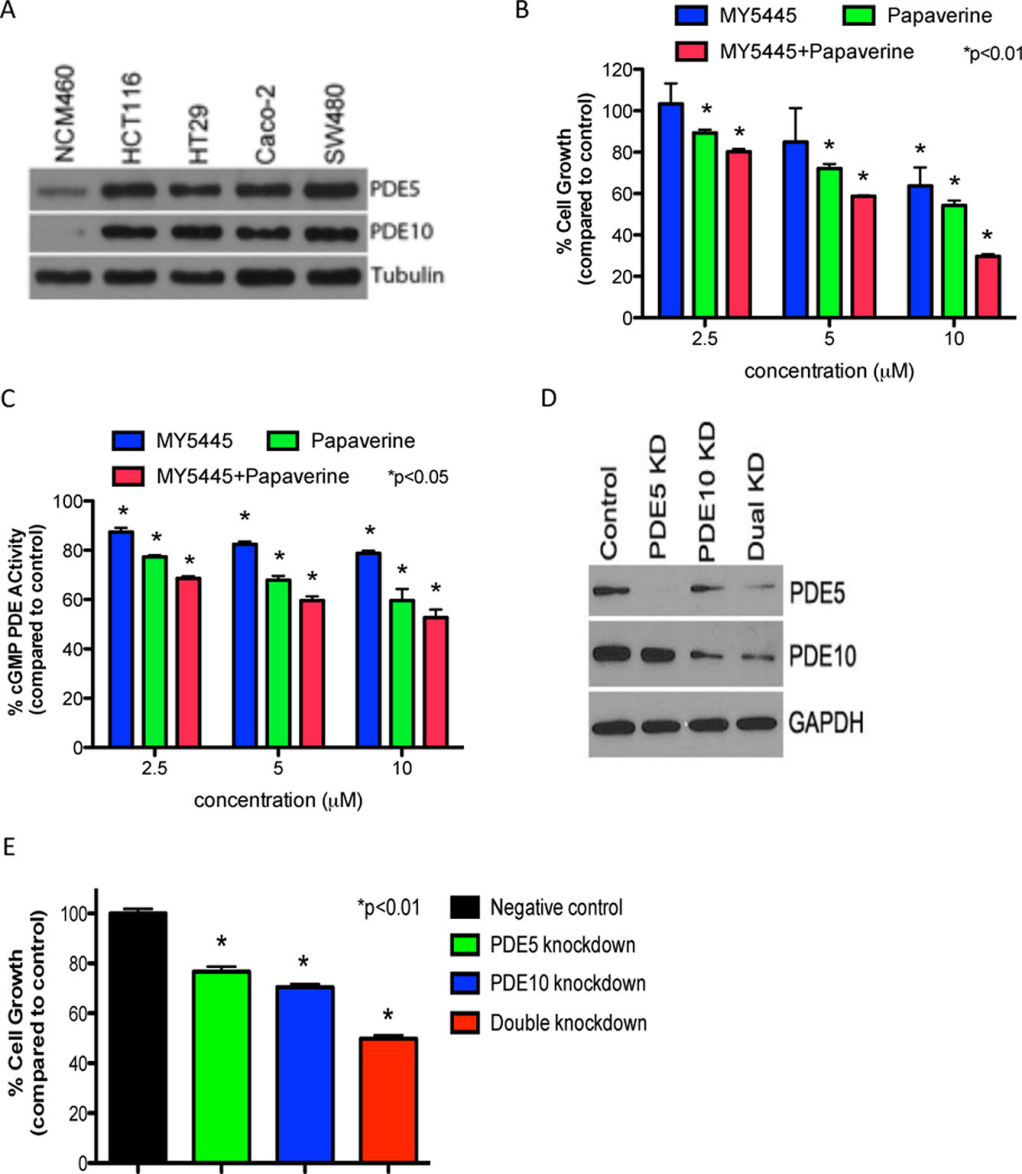
Dual inhibition of PDE5 and 10 results in greater inhibition of tumor cell growth than inhibiting either isozyme alone **A.** Expression of PDE5 and PDE10 in colon tumor cells and normal colonocytes. **B.** Inhibition of HCT116 colon tumor cell growth by the PDE5 inhibitor, MY5445, and the PDE10 inhibitor, papaverine, alone and in combination. **C.** Suppression of cGMP hydrolysis in HCT116 colon tumor cells by MY5445 and papaverine, alone and in combination. **D.** Confirmation of single knockdown of PDE5 or 10 and double knockdown of both isoyzmes in HT29 colon tumor cells by Western blotting. **E.** Effect of single PDE5 or 10 knockdown and double isozyme knockdown on human HT29 colon tumor cell growth.

Previous studies reporting the importance of PDE5 and 10 in regulating colon tumor cell growth [21, 22] call for further studies of these cGMP degrading isozymes in colon tumor cells. Western blotting using isozyme specific antibodies as shown in Figure 1A revealed that both PDE5 and PDE10 are elevated in human HT29, HCT116, SW480, and Caco-2 colon tumor cell lines compared with NCM460 normal colonocytes. As previously described, other cGMP degrading PDE isozymes, including PDE1, 2, 3, 9, and 11 were either not expressed or showed no difference in expression between colon tumor cells and colonocytes [21]. To determine if PDE5 and 10 are necessary for colon tumor cell growth, cells were treated with the PDE5 and 10 isozyme selective inhibitors, MY5445 and papaverine, respectively, alone and in combination. As shown in Figure 1B, both compounds effectively inhibit HCT116 colon tumor cell growth as single agents, while combined treatment caused greater inhibition than each agent alone. MY5445 and papaverine were confirmed to inhibited cGMP hydrolysis in lysates from HCT116 colon tumor cells within the same concentration range that inhibit growth and that combined treatment resulted in greater inhibition of enzymatic activity compared to either agent alone (Figure 1C). Consistent with low levels of PDE5 and PDE10 in colonocytes, MY5445 and papaverine did not significantly affect their growth as previously reported [14, 21].

To further define the roles of PDE5 and 10 in colon tumor cell growth, a double knockdown strategy was developed by transfecting stable PDE5 knockdown HT29 colon tumor cells with PDE10 siRNA. Single and dual knockdown of PDE5 and 10 were confirmed by Western blotting as shown in Figure 1D. Consistent with results observed with isozyme selective inhibitors, dual knockdown of PDE5 and 10 caused greater suppression of tumor cell growth compared with knockdown of either isozyme alone (Figure 1E). In addition, knockdown of PDE5 or PDE10 did not affect the growth of normal colonocytes as previously reported [21, 22].

### Design and synthesis of a novel sulindac derivative with dual PDE5 and 10 inhibitory activity

**Figure 2:**
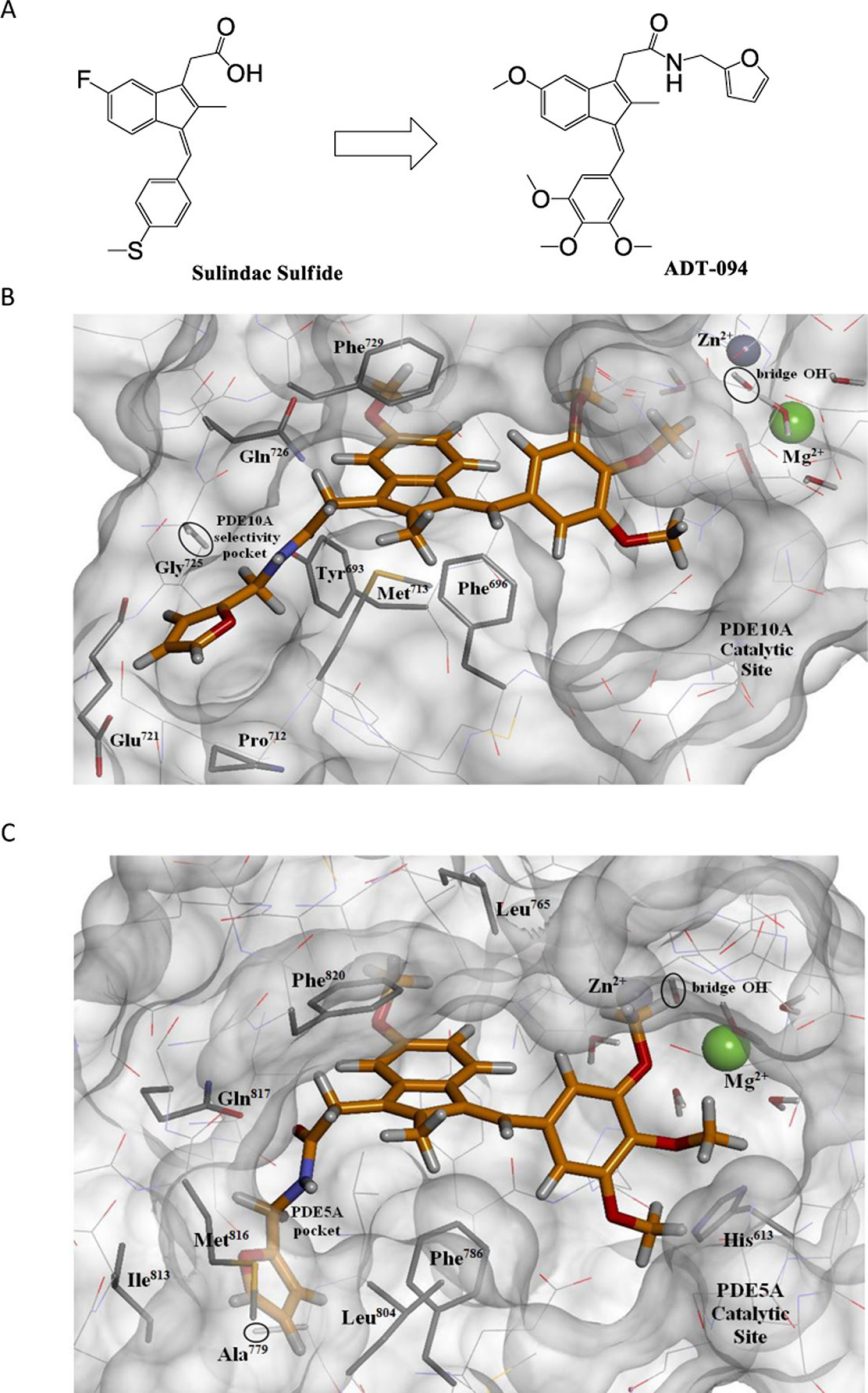
Chemical structure of ADT-094 and mode of binding to PDE5 and 10 **A.** chemical structure of SS and ADT-094. **B.** Optimized PDE10A+ADT-094 Complex. Carbon atoms of the inhibitor are in gold. The fused ring system of ADT-094 occupies the hydrophobic central region of the catalytic site directly below Phe^729^; the trimethoxyphenyl moiety is positioned near the Mg^2+^, Zn^2+^ system and its associated five waters and bridge hydroxide. The furan group in ADT-094 extends into the PDE10A selectivity pocket, a region bounded by Met^713^ and Pro^712^ and made accessible by the small size of Gly^725^. The furanic hydrogens interact favorably with both the carboxylate and carbonyl of Glu^721^. **C.** Optimized PDE5A+ADT-094 complex. Note that the fused ring system of ADT-094 occupies the central region of the PDE5A catalytic site below Phe^820^ and Leu^765^, and its methoxyphenyl group extends toward the Mg^2+^, Zn^2+^ system. The furan moiety is in a small pocket ringed by Ile^813^, Met^816^, Leu^804^, Phe^786^, and Ala^779^. This small pocket is proximal to and distinct from the selectivity pocket of PDE10A, which is occluded here by Met^816^, the counterpart of Gly^725^ in PDE10A. Instead, the PDE5A pocket is the consequence of the small size of Ala^779^.

Because PDE5 and 10 levels are elevated in colon tumor cells compared with colonocytes and the possibility that SS inhibits colon tumor cell growth by a mechanism involving PDE5 and/or 10 inhibition [13, 14, 19–22], we hypothesized that SS can be chemically modified to improve colon tumor cell growth activity by enhancing PDE5 and 10 inhibitory activity. A series of sulindac derivatives were therefore synthesized and screened for ability to inhibit PDE5 and 10 using recombinant enzymes. A lead compound referred to as ADT-094 was identified with potent colon tumor cell growth inhibitory activity in which the chemical structure is shown in Figure 2A. ADT-094 was derived from SS by substituting the carboxylic acid with an amide-linked furan moiety, the sulfide moiety to a trimethoxyphenyl group, and the fluoro group to a methoxy group.

Molecular modeling was used to analyze the mode of ADT-094 binding to the catalytic domains of PDE5 and 10. As shown in Figure 2B and 2C, the indene scaffold of ADT-094 occupies a hydrophobic pocket common to the catalytic domains in PDE5 and 10, which otherwise is occupied by the purine ring system of the cGMP/cAMP substrate [23–25]. The trimethoxyphenyl moiety of ADT-094 is positioned near the Mg^2+^, Zn^2+^ bimetal system, the site of cyclic phosphate hydrolysis. The PDE10 catalytic site is larger with more accessible space primarily in the distal region due to the smaller Thr^685^ and Thr^688^ versus Gln^775^ and Ile^778^ of PDE5. PDE10 also has a distal selectivity pocket [26] bound by Met^713^ and Pro^712^ and made accessible by the small size of Gly^725^ (Figure 2B). The furan moiety of ADT-094 extends into this selectivity pocket to achieve favorable interactions with both the carboxylate and carbonyl of Glu^721^. PDE5 has a distinct selectivity pocket as a consequence of the small size of Ala^779^ in the place of Tyr^693^ in PDE10 [27]. Met^816^ occludes the region characteristic of PDE10 and demarcates one side of the PDE5 selectivity pocket (Figure 2C). The amide linkage in ADT-094 is sufficiently flexible to permit a favorable placement of the furan group into the selectivity pocket of either isozyme.

**Figure 3:**
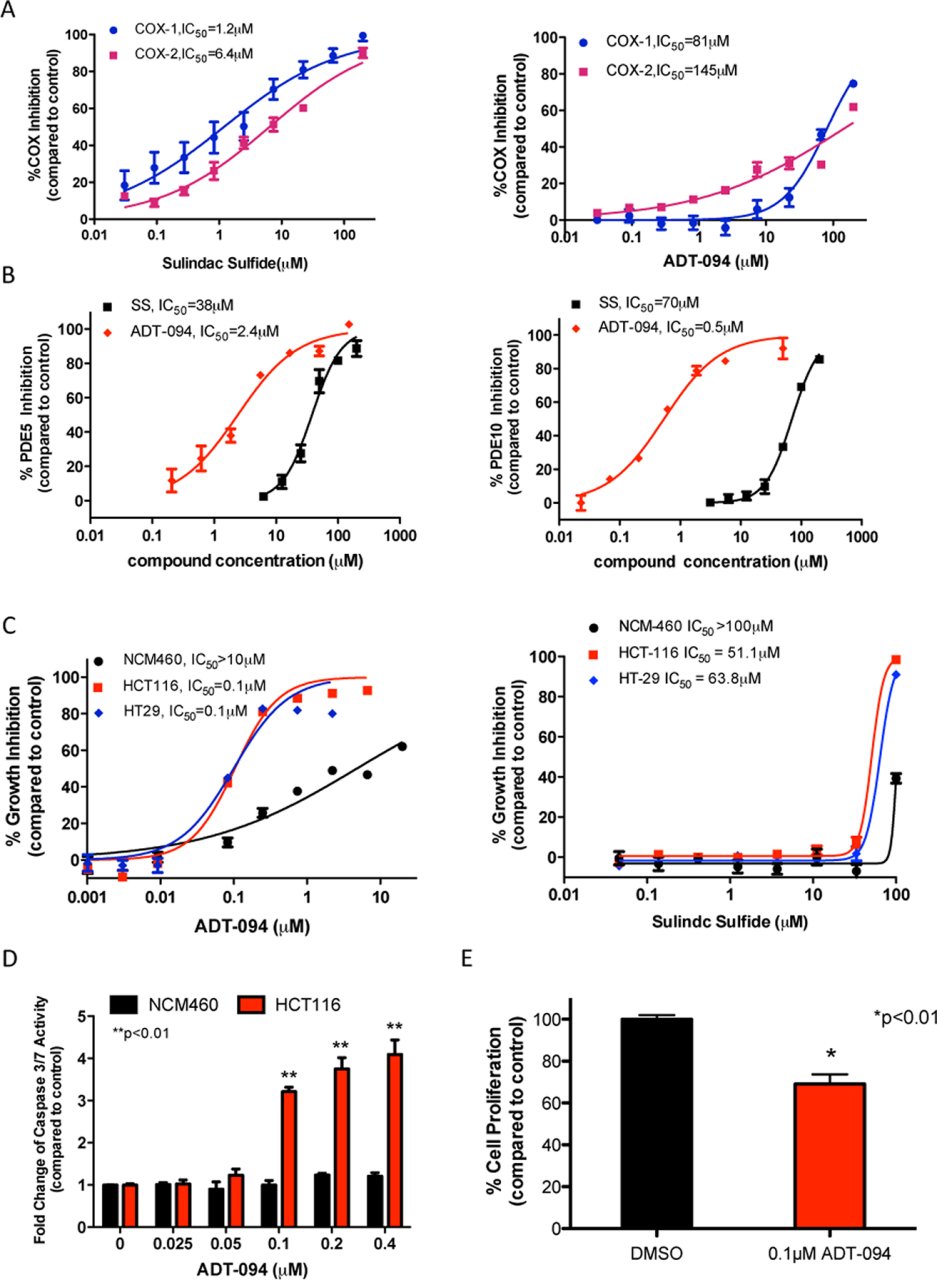
PDE and tumor cell growth inhibitory activity of ADT-094 **A.** COX-1 and COX-2 inhibitory activity of SS, but not ADT-094. **B.** Inhibition of PDE5 and PDE10 by SS and ADT-094. **C.** Tumor cell growth inhibitory activity of ADT-094 and SS as measured by luciferase-based ATP assay after 72 hours of treatment. **D.** Induction of apoptosis by ADT-094 as measured by activation of caspse-3 and -7 after 6 hours of treatment. **E.** Inhibition of proliferation after 24 hours treatment with 0.1 *μ*mol/L ADT-094 in HCT116 colon tumor cells as measured by EdU incorporation and flow cytometry.

Consistent with its known non-selective COX inhibitory activity, SS inhibits COX-1 and COX-2 with IC_50_ values of 1.2 and 6.4 *μ*mol/L, respectively. Likely as a result of substituting the carboxylic acid with a neutral group as previously reported [28], ADT-094 essentially lacks both COX-1 and COX-2 inhibitory activity (Figure 3A). Consistent with predictions from molecular modeling, ADT-094 more potently inhibits PDE5 and 10 with IC_50_ values of 2.4 and 0.5 *μ*mol/L compared with 38 and 70 *μ*mol/L for SS, respectively (Figure 3B). The improved PDE5 and 10 inhibitory activity of ADT-094 was paralleled with increased potency to inhibit the growth of human HCT116 and HT29 colon tumor cell lines with IC_50_ values of 0.1 *μ*mol/L, while SS inhibited growth with IC_50_ values of 51 and 68 *μ*mol/L, respectively (Figure 3C). ADT-094 also displayed a high degree of tumor cell selectivity in which normal colonocytes were essentially refractory to treatment. The tumor cell growth inhibitory activity of ADT-094 and its selectivity was associated with apoptotic cell death as evident by increased caspase activity in HCT116 colon tumor cells, but not in colonocytes (Figure 3D). In addition, ADT-094 inhibited the proliferation of HCT116 colon tumor cells at concentrations that suppress tumor cell growth and induce apoptosis (Figure 3E).

### ADT-094 selectively activates cGMP/PKG signaling in colon tumor cells

**Figure 4:**
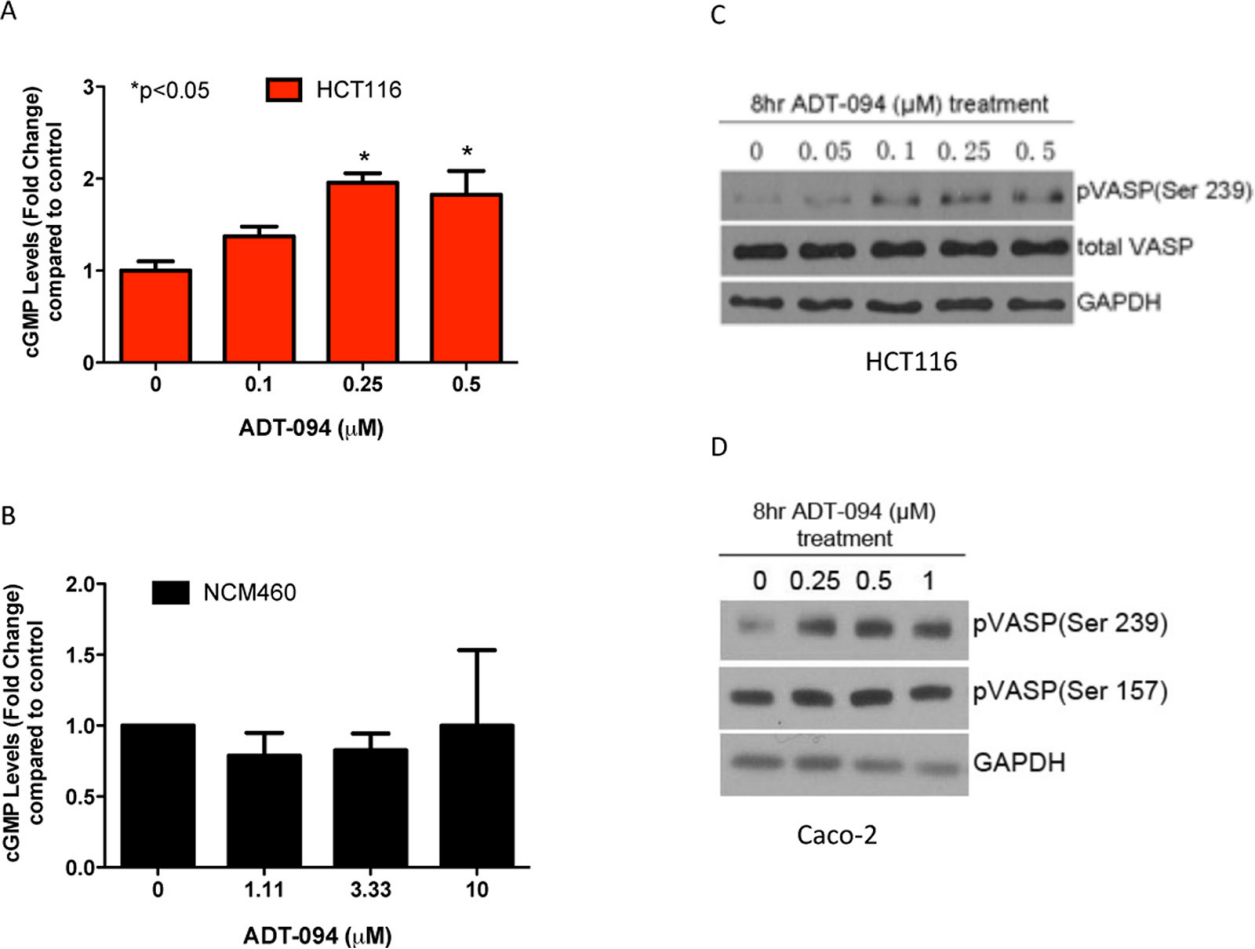
ADT-094 activates cGMP/PKG signaling pathway in colon tumor cells **A-B.** Dose-dependent increases in intracellular cGMP levels after 45 min of ADT-094 treatment in HCT116 colon tumor cells (A) but not in colonocytes (B). **C.** Dose-dependent increases in VASP phosphorylation at Ser^239^ after ADT-094 treatment in HCT116 colon tumor cells. **D.** Dose-dependent increases in VASP phosphorylation at Ser^239^ but not at Ser^157^ site after ADT-094 treatment in Caco-2 colon tumor cells.

To determine if the PDE5 and 10 inhibitory activity of ADT-094 is responsible for its tumor cell growth inhibitory activity, cGMP levels were measured in lysates from HCT116 colon tumor cells treated with ADT-094 at concentrations that suppress tumor cell growth. As shown in Figure 4A, ADT-094 treatment significantly increased cGMP levels within the same concentration range as required for tumor cell growth inhibition. Consistent with its tumor cell selectivity, ADT-094 did not significantly affect intracellular cGMP levels in normal colonocytes (Figure 4B).

To determine if cGMP elevation by ADT-094 is sufficient to activate cGMP-dependent protein kinase G (PKG), treatment effects on the phosphorylation of vasodilator-stimulated phosphoprotein (VASP) were determined in colon tumor cells treated with ADT-094. As previously reported, VASP is preferentially phosphorylated at serine 239 residue by PKG in which the phosphorylation of this amino acid serves as an indicator of PKG activity [29]. As shown in Figure 4C, ADT-094 increased levels of phospho-Ser239 VASP in HCT116 colon tumor cells without affecting total VASP levels. These data also provide evidence that ADT-094 activates PKG at concentrations that parallel those that inhibit colon tumor cell growth and increase intracellular cGMP levels. We also determined the effect of ADT-094 on cAMP signaling by measuring cAMP-dependent protein kinase (PKA) activity following treatment. PKA preferentially phosphorylates VASP at serine 157 residue as previously reported and is used as an indicator of PKA activity [30]. ADT-094 did not affect PKA activity at concentrations that cause PKG activation in Caco-2 colon tumor cells (Figure 4D). These data provide evidence that PKG activation occurs in response to ADT-094 treatment at concentrations that parallel those required for tumor cell growth inhibition.

### Dual inhibition of PDE5 and 10 suppresses β-catenin/TCF transcriptional activity

**Figure 5:**
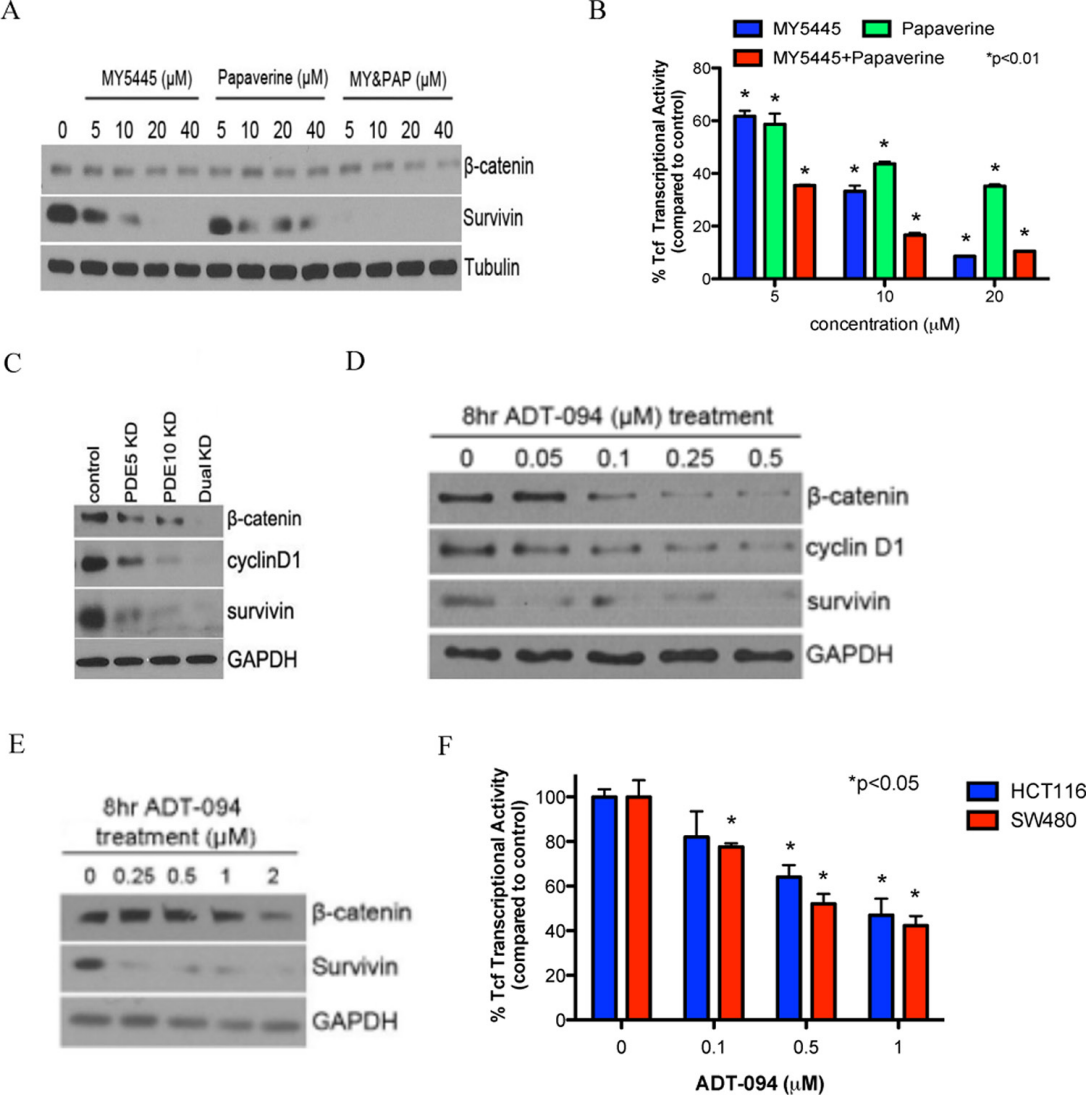
Inhibition of PDE5 and 10 by specific inhibitors or ADT-094 can attenuate β-catenin signaling **A.** Inhibition of expression of β-catenin and its regulated survivin by the MY5445, papaverine, alone and in combination in HCT116 cells. **B.** Suppression of β-catenin/TCF transcriptional activity followed by 24 hours of treatment with MY5445, papaverine, alone and in combination in HCT116 cells. **C.** Inhibition of Wnt/β-catenin signaling by knockdown of PDE5 or 10 alone and dual isozymes knockdown in HT29 cells. **D** and **E.** Inhibition of expression of β-catenin and its regulated cyclin D1 and survivin in HCT116 (D) and Caco-2 (E) colon tumor cells after ADT-094 treatment. **F.** β-catenin/TCF transcriptional activity in HCT116 and SW480 cells followed by 24 hours of ADT-094 treatment.

As previously reported, SS treatment can inhibit Wnt/β-catenin signaling to attenuate TCF transcriptional activity [12, 20, 31–33], which is particularly significant for its antineoplastic activity given that stabilized β-catenin is associated with colorectal tumorigenesis. Consistent with a role of cGMP/PKG in regulating Wnt/β-catenin signaling as reported previously [21, 22, 31], MY5445 and papaverine reduced β-catenin levels, TCF transcriptional activity, and the expression of survivin in HCT116 colon tumor cells at concentrations that parallel those required for PKG activation (Figure 5A, 5B). Consistent with results from growth assays, combined treatment with MY5445 and papaverine caused a greater inhibitory effect on β-catenin/TCF transcriptional activity compared with each agent alone. The ability of PDE5 and 10 inhibitors to suppress β-catenin/TCF transcriptional activity was confirmed by the double knockdown strategy as described above (Figure 5C).

To determine if ADT-094 can suppress Wnt/β-catenin signaling by a mechanism involving PKG activation, PKG activation and β-catenin levels were measured in the same lysates from colon tumor cells treated with ADT-094. As shown in Figure 5D and 5E, ADT-094 caused a dose-dependent decrease in β-catenin levels that parallel concentrations that are required to activate PKG. Levels of cyclin D1 and survivin, which are under the control of the β-catenin/TCF complex [34] were also decreased. Furthermore, ADT-094 treatment significantly inhibited β-catenin/TCF-mediated transcription in HCT116 and SW480 colon tumor cells as measured by a luciferase reporter assay (Figure 5F).

## DISCUSSION

Despite the promising cancer chemopreventive activity of NSAIDs as evident by epidemiological and preclinical studies, this class of drugs is not recommended for long-term use in humans due to the potential for toxicities associated with COX inhibition and the suppression of physiological prostaglandins. However, numerous investigators have concluded that the basis for the antineoplastic activity of NSAIDs may not require COX inhibition, which suggests the feasibility of developing safer and possibly more efficacious derivatives by targeting the underlying mechanism. We have previously reported that cGMP PDE inhibition and activation of cGMP/PKG pathway is closely associated with the tumor cell growth inhibitory and apoptosis inducing activity of SS [12, 14, 20, 33, 35]. SS inhibits cGMP hydrolysis by PDE2, 3, 5, and 10 with IC_50_ values that parallel those for colon tumor cell growth inhibition, but does not interfere with cAMP hydrolysis by any PDE isozyme. In addition, activators of cAMP (e.g. PDE4 inhibitors) or PKA (e.g. forskolin) do not inhibit colon tumor cell growth, which lead us to hypothesize that inhibition of cGMP degrading PDE isozymes are responsible for the tumor cell growth inhibitory activity of SS. Here we show that PDE5 and 10 are overexpressed in colon tumor cell lines compared with normal colonocytes and that inhibition with small molecule inhibitors or siRNA selectively suppress colon tumor cell growth. The mechanism involves cGMP elevation and activation of PKG to inhibit β-catenin/TCF transcriptional activity, which is aberrantly induced during colorectal cancer [13, 14, 19–21]. We report for the first time that inhibition of PDE5 and 10 results in greater growth suppression than inhibiting either isozyme alone. A novel, non-COX inhibitory sulindac derivative, ADT-094, also was characterized in this study to have >500 fold higher potency than SS to inhibit colon tumor cell growth with improved tumor cell selectivity. ADT-094 suppresses tumor cell growth by inhibiting PDE5 and 10 to activate cGMP/PKG signaling, leading to the disruption of β-catenin/TCF transcriptional activity and the synthesis of critical proteins required for the proliferation and survival of tumor cells.

We previously reported that modification of the carboxylic acid moiety on SS with a positively charged moiety can effectively block binding to both COX-1 and -2 binding [20, 36]. This effect is further demonstrated by ADT-094 in which a neutral furan moiety is substituted for the carboxylic acid, which effectively blocked COX-1 and -2 binding. These observations are consistent with studies by others who have also concluded that a COX-independent mechanism is fully or partially responsible for the antineoplastic activity of various anti-inflammatory drugs belonging to chemically distinct families [37–39]. For example, the rank order potency among NSAIDs to inhibit prostaglandin synthesis and growth of tumor cells do not correlate in which higher doses are generally required to inhibit tumor cell growth compared with concentrations needed to inhibit COX activity [8, 40].

Despite lacking COX inhibitory activity, ADT-094 inhibits colon tumor cell growth with IC_50_ values at least 500 fold less than SS. The improved potency to inhibit colon tumor cell growth was paralleled with an increase in potency to inhibit PDE5 and 10. The ability of isozyme-selective inhibitors of PDE5 and 10, as well as siRNA knockdown to selectively suppress colon tumor cell growth in a manner similar to ADT-094 suggest that inhibition of both isozymes mediates the growth inhibitory activity of ADT-094 [20].

The ability of ADT-094 to selectively inhibit colon tumor cell growth and induce apoptosis was associated with increased expression of PDE5 and 10 in colon tumor cells compared with colonocytes. The elevation of PDE5 and 10 in colon tumor cells is consistent with previous studies reporting that PDE5 and 10 are overexpressed in human colon adenomas and adenocarcinomas compared with normal colonic epithelium as determined by immunohistochemistry and other methods [14, 21].

ADT-094 inhibition of colon tumor cell growth is associated with inhibition of proliferation and induction of apoptosis, which occurred at concentrations comparable with those necessary for cGMP elevation and PKG activation. Although PDE10 is a dual-substrate isozyme, ADT-094 displays a selective effect on activating PKG without affecting PKA activity at concentrations that cause growth inhibition of colon tumor cells. These results are consistent with previous studies reporting that siRNA knockdown of PDE10 selectively inhibits cGMP hydrolysis without affecting cAMP hydrolysis in colon tumor cells [21]. This may be attributed to an abundance of cAMP degrading isozymes (e.g. PDE4) known to be expressed in colon tumor cells [12] that could compensate for the effects of PDE10 inhibition or knockdown on cAMP signaling. Alternatively, PDE10 is known to have higher affinity for cAMP (50 nmol/L) than for cGMP (3 μmol/L), but a five-fold lower Vmax for cAMP compared with cGMP [41–43]. Therefore, PDE10 may function in cells as a cAMP-inhibited cGMP PDE. However, further studies are necessary to determine if ADT-094 has selective effects on cGMP signaling that distinguish it from other PDE10 inhibitors.

Of relevance to our findings of antitumor effect of the cGMP/PKG pathway, other investigators have shown the association between cGMP signaling and colorectal tumorigenesis. For example, a previous study has shown that guanylyl cyclase (GC)-deficient mice display increased susceptibility to colon tumorigenesis [44]. In addition, the GC agonist, uroguanylin, inhibits tumor formation in the *Apc*^Min/+^ mouse model and increases rates of apoptosis within the tumors [45]. Other studies indicate that human colon tumor cells transfected with constitutively active mutants of PKG can undergo apoptosis and are unable to form colonies [46]. PKG is also down-regulated in many cancer types including colorectal cancer, and is important for tumor angiogenesis [47].

We also investigated the potential involvement of Wnt/β-catenin signaling in the antineoplastic activity of ADT-094, given that aberrant up-regulation of this pathway is common in colorectal cancer patients [48, 49] and suppression of β-catenin expression and β-catenin/TCF transcriptional activity has been previously implicated in the anticancer activity of sulindac [12, 14, 20, 31, 50]. We provide here the first evidence that suppression of both PDE5 and 10 causes an additive inhibitory effect on Wnt/β-catenin signaling as compared to inhibiting either isozyme individually. Furthermore, ADT-094 treatment can inhibit β-catenin levels and attenuate β-catenin/TCF-mediated transcription. These observations are consistent with reports from other investigators showing that sulindac metabolites or activators of the cGMP/PKG pathway can suppress the oncogenic activity of β-catenin [12, 14, 31, 33, 51–53].

The mechanism by which ADT-094 treatment suppresses the oncogenic activity of β-catenin may be at the transcriptional level given that β-catenin mRNA levels and CTNNB1 promoter activity were reduced in colon tumor cells following knockdown of PDE5 or PDE10 or by SS treatment [21, 22]. Our observations are consistent with reports that show activation of PKG can suppress β-catenin transcription [31], although further studies are necessary to understand the underlying mechanism by which PKG inhibits transcription of β-catenin.

In conclusion, dual inhibition of PDE5 and 10 can suppress colon tumor cell growth through a mechanism involving elevation of intracellular cGMP levels, activation of PKG, and attenuation of β-catenin-dependent TCF transcriptional activity to inhibit proliferation and induce apoptosis. These findings are significant given the overexpression of PDE5 and 10 in colon tumor cells that suggest deficiencies in the cGMP/PKG pathway play an unrecognized role in tumorigenesis. The selective nature by which inhibitors suppress tumor cell growth also suggest the feasibility of targeting PDE5 and 10 for cancer drug discovery, although further studies are necessary to determine if there are efficacy or safety advantages in targeting specific isozymes.

## MATERIALS AND METHODS

### Drugs and reagents

Sulindac sulfide (SS) and papaverine were purchased from Sigma-Aldrich, while 1-(3-chlorophenylamino)-4-phenylphthalazine (MY5445) was purchased from BioMol. Recombinant PDE isozymes were purchased from BPS Biosciences. The PDE10 antibody was purchased from GeneTex, while the PDE5 antibody was purchased from Cell Signaling Technologies. Non-targeting control siRNA and PDE10 specific siRNAs were purchased from Qiagen. RNAiMAX transfection reagent was purchased from Invitrogen. DMSO was used as vehicle for all compounds unless otherwise noted. All other reagents were purchased from Sigma.

### Chemical synthesis

ADT-094 was synthesized starting from 4-anisobenzaldehyde. The intermediate product, 6-methoxy-2-methyl-2, 3-dihydro-1H-inden-1-one was treated with cyanoacetic acid in the presence of ammonium chloride and acetic acid to generate 2-(5-methoxy-2-methyl-1H-inden-3-yl) acetonitrile. Hydrolysis of the nitrile gave the 2-(5-methoxy-2-methyl-1H-inden-3-yl) acetic acid. Reacting of the acid with 3, 4, 5-trimethoxybenzaldehyde in the presence of sodium methoxide in methanol resulted in (Z)-2-(5-methoxy-2-methyl-1-(3, 4, 5-trimethoxybenzylidene)-1H-inden-3-yl) acetic acid as a yellow solid. ADT-094 ((Z)-N-(furan-2-ylmethyl)-2-(5-methoxy-2-methyl-1-(3, 4, 5-trimethoxybenzylidene)-1H-inden-3-yl) acetamide) was obtained by an amide coupling reaction catalyzed by CDI. The final product was characterized by proton magnetic resonance spectroscopy, electro-spray mass spectrometry, and elemental analysis before biological testing.

### Cells and cell culture

Human colon tumor cell lines, HCT116, HT29, SW480 and Caco-2 were obtained from the American Type Culture Collection (ATCC) and grown under standard cell culture conditions in RPMI 1640 medium containing 5% serum at 37°C in a humidified atmosphere with 5% CO_2_. The human colonocyte line, NCM460, that is derived from normal human colon mucosa [54] was obtained from INCELL and grown in INCELL’s enriched M3:10 medium with 10% serum as recommended by supplier. All cell lines were expanded upon delivery, and numerous aliquots of low passage cells were preserved in liquid N_2_. Cells were passaged no longer than 2 months. Tumor cell lines obtained from ATCC were characterized by STR profiling as performed by ATCC. The NCM460 line was characterized by INCELL as described previously (e.g. tumorigenicity testing) [54]. No additional re-authentication of the cell lines was performed except for experimental reasons (e.g. confirmation of cell doubling time, morphology, sensitivity to SS, PDE5/10 expression levels, etc).

### siRNA-mediated knockdown

The PDE10 siRNA target sequence was 5′-GACCGGATCACTAAACCTTAA-3′. For single PDE10 knockdown or PDE5/10 double knockdown study, siRNA duplexes were transfected into vector control HT29 cells or stable PDE5 knockdown HT29 cells using RNAiMAX transfection reagent according to manufacturers’ specifications and incubated at 37°C for 72 hours.

### Generation of stable PDE5 knockdown cell line

The stable PDE5 knockdown and vector control HT29 cells were generated as described previously [55]. Briefly, The PDE5 siRNA target sequence was 5′-ATGGAACAAAGGCATTGTGGG-3′. The negative control siRNA sequence was 5′-AAGCGTGGCTGGATGATCACC-3′. The retroviral supernatant of siRNA from Phoenix-Ampho packaging cells was incubated with HT29 cells with 5 μl of polybrene for 24 hours. The stably transfected PDE5 siRNA cells were selected in 10 μg/ml puromycin for 72 hours before PDE5 activity, mRNA, and protein levels were evaluated.

### COX assay

COX-1 and COX-2 activities were measured using purified ovine COX-1 and COX-2 with colorimetric assay kits obtained from Cayman as previously reported [36]. The activities of COX-1 and COX-2 were measured after the addition of arachidonic acid and incubation at 25°C for 5 min by absorbance at 590 nm as specified by the manufacturer.

### Growth assay

Cells were plated in 96-well microtiter plates at a density of 5,000 cells per well. For drug treatment, cells were treated with compound or vehicle, and incubated at 37*°*C for 72 hours. All growth assays were performed in 5% serum unless otherwise noted. Deionized water was used as vehicle control for papaverine. For siRNA assays, cells were transfected with siRNA under the same condition described above. The effect of treatment on cell growth was measured using the Cell Titer Glo Assay as specified by the manufacturer (Promega).

### Apoptosis assay

Cells were plated in 96-well microtiter plates at a density of 10,000 cells per well, and allowed to attach overnight. Cells were treated with ADT-094 or vehicle, and incubated at 37°C for 6 hours. All apoptosis assays were done in 5% serum using either tumor cells or normal colonocytes. The induction of apoptosis caused by treatment was determined using Caspase 3/7 Glo Assay (Promega), which is a luminescent assay that measures substrate cleavage by caspases-3 and -7. The assay was done according to manufacturer’s specifications.

### Cell proliferation assay

The antiproliferative activity of ADT-094 was determined by measuring EdU (5-ethynyl-2′-deoxyuridine) incorporation during DNA synthesis. Cells were plated at a density of 1.5 × 10^6^ cells per 10 cm tissue culture dish and incubated overnight at 37*°*C. After growing the cells in serum-free media overnight, the cells were treated with ADT-094 or vehicle in medium supplemented with 10% serum for 6 hours. A final concentration of 10 *μ*mol/L EdU was added to each dish and incubated for an additional 18 hours. Cells were harvested and analyzed using the Click-iT EdU Alexa Fluor 488 Proliferation Assay (Invitrogen) according to the manufacturer’s specifications. The percentage of proliferating cells was quantified using a BD FACSCalibur flow cytometer.

### PDE assay

PDE activity was measured using the IMAP fluorescence polarization (FP) PDE assay (Molecular Devices) in which binding of hydrolyzed fluorescent cyclic nucleotide substrate to the IMAP reagent increases FP as described previously [13].

### cGMP assay

Cells were plated at a density of 1 × 10^6^ cells per 10 cm tissue culture dish, incubated for 48 hours, and treated with ADT-094 or vehicle control. After 45 min of treatment, cells were lysed and assayed for cGMP content using the cGMP Direct Biotrak EIA kit (GE Healthcare Life Sciences). The assay was done according to the manufacturer’s specifications.

### Western blotting

Western blotting was performed as described previously [13]. Cells were treated with ADT-094 or vehicle for 8 hours before cell lysates were collected.

### Luciferase reporter assay

Cells were seeded at a density of 5 × 10^4^ cells per well in 24-well tissue culture plates and incubated overnight at 37*°*C. Cells were transiently transfected with 0.1 *μ*g TOP-FLASH constructs (Millipore) and 0.1 μg β-galactosidase-expressing vector (Promega). After 24 hours of transfection, cells were treated with ADT-094 or vehicle for 24 hours. At the end of treatment, cells were lysed and both luciferase and β-galactosidase activities were measured using kits from Promega. All luciferase activity was normalized to β-galactosidase activity.

### Quantum-based computational modeling of PDE5A/10A+ADT-094 complexes

Molecular modeling was performed using the valence-electron semi-empirical PM6 model [56] as implemented in the Gaussian09 suite of quantum chemistry programs (Gaussian, Inc., Rev. C.01). A charge-neutral model of the catalytic site of PDE10A was built from pdb entry 2OUN (PDE10A+AMP) by selection of 53 residues defining the region. Breaks in the peptide backbone were terminated with unprotonated amine and aldehyde functional groups. The bound AMP ligand was removed, and waters of hydration and a bridge hydroxide were inserted into the Mg^2+^, Zn^2+^ system; the proximal His^525^ was protonated, in accord with the results of an earlier quantum-based study [25]. His^525^ belongs to a PDE-conserved His/Glu tandem, which serves to both prepare the bridge hydroxide for nucleophilic attack of the cGMP (cAMP) phosphate diester and to donate a proton to the GMP (AMP) hydrolyzed product [23, 25]. Other residues were charged appropriately for pH = 7. PM6 optimization was carried out to minimize the positions of all added hydrogen atoms. By the same protocol, a 50-residue model of the PDE5A catalytic site was built from pdb entry 1TBF (PDE5A + Viagra). ADT-094 was manually docked within the PDE5A catalytic site in 7 distinct initial orientations. Partial PM6 optimizations were then carried out in which the inhibitor was free to move.

### Experimental design and statistical analysis

The IC_50_ values were determined as described previously [13]. All experiments were repeated a minimum of three times to determine the reproducibility of the results. All error bars represent standard error of the mean (SEM). Statistical analysis of differences between samples was performed using the Student’s *t*-test. A *P* value of < 0.05 was considered statistically significant.
